# Assessing and Comparing Global Health Competencies in Rehabilitation Students

**DOI:** 10.1155/2013/208187

**Published:** 2013-12-07

**Authors:** Mirella Veras, Kevin Pottie, Debra Cameron, Govinda P. Dahal, Vivian Welch, Tim Ramsay, Peter Tugwell

**Affiliations:** ^1^University of Ottawa/Saint Elizabeth Health Care, Nursing Best Practice Research Center, University of Ottawa, 1118E, 451 Smyth Road, Ottawa, ON, Canada K1H 8M5; ^2^Departments of Family Medicine and Epidemiology and Community Medicine, University of Ottawa, 1 Stewart Street, Ottawa, ON, Canada K1N 6H7; ^3^Department of Occupational Science and Occupational Therapy, University of Toronto, Room 160, 500 University Avenue, Toronto, ON, Canada M5G 1V7; ^4^Institute of Population Health, Faculty of Medicine, University of Ottawa, 1 Stewart Street, Ottawa, ON, Canada K1N 6H7; ^5^Canada Foundation for Nepal, Canada; ^6^Bruyère Research Institute, University of Ottawa, 1 Stewart Street, Ottawa, ON, Canada K1N 6H7; ^7^Ottawa Hospital Research Institute, Clinical Epidemiology Program, 1 Stewart Street, Ottawa, ON, Canada K1N 6H7; ^8^Departments of Family Medicine and Epidemiology and Community Medicine, 1 Stewart Street, Ottawa, ON, Canada K1N 6H7

## Abstract

*Purpose*. Globalization is contributing to changes in health outcomes and healthcare use in many ways, including health professionals' practices. The objective of this study was to assess and compare global health competencies in rehabilitation students. *Method*. Online cross-sectional survey of physiotherapy and occupational therapy students from five universities within Ontario. We used descriptive statistics to analyze students' perceived knowledge, skills, and learning needs in global health. We used Chi-square tests, with significance set at *P* < 0.05, to compare results across professions. *Results*. One hundred and sixty-six students completed the survey. In general, both physiotherapy and occupational therapy students scored higher on the “relationship between work and health,” “relationship between income and health,” and “socioeconomic position (SEP) and impact on health” and lower on “Access to healthcare for low income nations,” “mechanisms for why racial and ethnic disparities exist,” and “racial stereotyping and medical decision making.” Occupational therapy students placed greater importance on learning concerning social determinants of health (*P* = 0.03). *Conclusion*. This paper highlights several opportunities for improvement in global health education for rehabilitation students. Educators and professionals should consider developing strategies to address these needs and provide more global health opportunities in rehabilitation training programs.

## 1. Introduction

Globalization has become a key word in the 21st century and is defined here as “the ways in which nations, businesses, and people are becoming more connected and interdependent across national borders through increased economic integration, communication, cultural diffusion, and travel” [1]. Indeed, globalization has influenced health determinants and changed health outcomes [1]. The social determinants of health (SDH), including health services, are influenced by the distribution of money, power, and resources at global, national, and local levels [2–4]. In addition, natural disasters such as floods, earthquakes, and volcanic eruptions as well as complex emergencies including war, civil strife, and food shortages are now considered global problems which have a considerable impact on population health worldwide [5].

Evidence shows that such calamities have progressively increased the number of deaths, illnesses, and disabilities and, therefore, the economic costs of treatment and rehabilitation [5]. For instance, the earthquake that occurred in Haiti in 2010 resulted in extensive infrastructure damage, and it was estimated that there were 220 000 casualties and more than 300 000 people suffered from injuries including fractures, burns, amputations, and brain and spinal cord injuries [6, 7]. Additionally, there is an increase in the number of people with chronic diseases in both developed and developing countries, which may lead to their development of several disabilities [5]. These health issues highlight the role that rehabilitation professionals, such as occupational therapists and physiotherapists, play in improving daily living and functional skills in people who have suffered from exposure to natural disasters and chronic conditions and their implication on global health [7].

Presently, there is an extraordinary interest in global health among students, health professionals, and educators [8]. Some universities have departments or units that focus on global health and offer opportunities, specifically, to physiotherapists and occupational therapists to work overseas [9, 10]. For example, The International Centre for Disability and Rehabilitation (ICDR) at the University of Toronto has encouraged a focus on global health issues related to disability and rehabilitation within the rehabilitation sciences. The numbers of students who have had the opportunity to experience an international clinical internship has increased significantly in the past ten years [11].

Occupational therapists and physiotherapists have been participating in paid and unpaid work overseas as well as international internships as part of their educational programs [7, 12]. The World Confederation for Physical Therapy (WCPT) carries out several programs and projects for physiotherapists working overseas as well as supporting international campaigns to endorse the contribution of the profession for global health [13]. The president of the World Federation for Occupational Therapists (WFOT) emphasizes the commitment of the profession to contribute to global health and the World Health Organization (WHO) initiatives, which includes several topics for the global health agenda: mental health, human resource planning, healthy aging, actions for health and well-being, and disability [14].

Research on disability and health care suggests that people with impairments face noteworthy personal and cultural barriers to access healthcare facilities. The personal barriers are related to transportation, communication, finance, and insurance. The cultural barriers include misconceptions about people with disabilities, lack of respect, and reluctance to provide care for these populations [15]. Although there is an increased interest in global health, little is known about the global health competencies of occupational therapy and physiotherapy students. Hence, the purpose of this study is to assess the knowledge, skills, and learning needs related to global health and health equity of occupational therapists and physiotherapists students in Ontario, Canada.

## 2. Methods

An online cross-sectional survey was administered using SurveyMonkey online survey to physiotherapy and occupational therapy students registered in five universities in Ontario.

### 2.1. Instrument

A forty-seven-item online global health education survey was firstly developed to gather information regarding self-perceived knowledge, skills, and learning needs in global health. The survey was developed by adapting three other instruments: (1) resident physicians' knowledge of underserved patients, a validated survey used to measure actual and perceived resident physician's knowledge of underserved patient populations in the United States done by Wieland and adapted by the research team for the Canadian population (17 items) [16]; (2) a global health competency skills survey for medical students by Augustincic (14 items) [17]; (3) the Canadian Medical Education Directives for Specialists (CanMEDS) competencies as [18]. The survey consisted of questions subdivided into four parts: (1) knowledge in global health and health equity (3-point scale); (2) global health skills (5-point scale); (3) learning needs about global health (16 items) (5-point scale); and (4) about you: demographic and socioeconomic questions. This global health education survey was described previously and demonstrates good internal consistency with a Cronbach's alpha > 0.8 [19]. The full analysis of the global health education survey was described in a previous publication [20].

The questions regarding self-assessed confidence in global health (part 01/04) asked respondents whether they felt “not at all confident,” “somewhat confident,” or “very confident.” The questions received the following code: 0 (not at all), 0.5 (somewhat), and 1 (very). Self-perceived skills in global health (part 2/4) could be answered by either “strongly agree,” “agree,” “neutral,” “disagree,” or “strongly disagree.” The questions received codes varying between 0 and 1 (item scale [0-1]—for negative questions: 1 = strongly disagree, 0.75 = disagree, 0.50 = neutral, 0.25 = agree, and 0 = strongly agree; for positive questions: 1 = strongly agree, 0.75 = agree, 0.50 = neutral, 0.25 = disagree, and 0 = strongly disagree). Therefore, by averaging all respondents' answers to a given question and multiplying that average by 100, each question was given a number between 0 and 100. The score 0 represented a complete lack of confidence and 100 represented feeling completely confident. Learning needs in global health (part 3/4) could be answered by either “not at all important,” “somewhat important,” “neutral,” “important,” “very important,” or “extremely important.” The fourth part of the survey included demographic questions.

### 2.2. Participants

Students from five universities, within Ontario, Canada, were invited to participate in the study. Inclusion criteria were predefined as follows: 18 years or older or 1st year student from a master's program in physiotherapy or occupational therapy program in one of the five participating universities in Ontario.

### 2.3. Data Collection

From May to October 2011, directors or coordinators of physiotherapy and occupational therapy programs were contacted to collaborate the survey. They were asked to send an e-mail invitation to all physiotherapy and occupational therapy students to invite the prospects to participate in the survey. Then, an e-mail containing a hyperlink to the survey and a consent form was sent to all students. Two reminder e-mails were sent at one and two week intervals.

### 2.4. Ethical Considerations

Ethical approval was obtained for this study from the Ottawa Hospital Research Ethics Board, the University of Ottawa, and the University of Western Ontario.

## 3. Results

The response rate was 23.7% and thus a total of 166 participants were included in the following analysis. Most of the participants were females, originally from Canada, from a higher socioeconomic background and were able to speak at least two languages.

### 3.1. Participant's Characteristics

All five eligible universities which offered both physiotherapy and occupational therapy programs in Ontario, Canada, were represented in this study.  Table 1 shows the demographic characteristics of respondents. Most of the participants in both programs were female (81% physiotherapy students (PTS) and 95% occupational therapy students (OTS)). The majority of the respondents were born in Canada (87% physiotherapy students and 89% occupational therapy students). The physiotherapy participants were older (mean age = 26.47 years) than occupational therapy students (mean age = 24.91 years). The majority of participants were from a white family background (77% physiotherapy students and 80% occupational therapy students). Slightly more than one third of interviewed students of both categories had parents with an annual income of CAN$ 80,000 or greater. Physiotherapy students had relatively higher multilingual abilities compared to their counterparts from occupational therapy (Table 1).

### 3.2. Self-Perceived Global Health Knowledge Scores

Students were asked to rate their self-perceived knowledge in several global health and health equity topics. The self-perceived knowledge scores of physiotherapy and occupational therapy students in twelve domains of global health are presented in Table 2. Self-perceived knowledge of physiotherapy students was found to be highest (71%) in “relationship between income and health” followed by “relationship between work and health” (62%) and “language barrier and adverse impact on health and health care” (66%). Similarly, self-perceived knowledge of occupational therapy students was highest in “relationship between work and health” (82%) domain followed by “relationship between income and health” (77%) and “socioeconomic position (SEP) and impact on health” (68%) (Table 2).

### 3.3. Self-Perceived Global Health Skills for Physiotherapy and Occupational Therapy Students Guided by the CanMEDS Framework

The scores of perceived global health skills of physiotherapy and occupational therapy students in eleven skills domains guided by the CanMEDS framework are presented in Table 3. Both physiotherapy and occupational therapy students perceived themselves to have higher global health skills in (i) listening (PS = 76% and OTS = 73%), (ii) clinical competency (PS = 70% and OTS = 67%), and (iii) identifying needs (PS = 63% and OTS = 57%) and having lower skills in (iv) being active in global health (PS = 33% and OTS = 33%) domains.

### 3.4. Learning Needs in Global Health

Learning needs in global health for physiotherapy and occupational therapy students are presented in Table 4. Participants considered it “extremely important” to learn about the topic “understand the relationship between health and human rights” and “not at all important” to learn about the “relationship between access to clean water, sanitation, and nutrition on individual and population health.” Statistically significant relationships between variables considered for learning needs in global health were assessed using chi-square test. The results show that there was a significant result for the following topics: “relationship between health and social determinants of health and how social determinants vary across world regions” (*P* = 0.03) and “relationship between access to clean water, sanitation, and nutrition on individual and population health” (*P* = 0.03).

Almost 70% of the occupational therapy students reported that it is extremely important to learn about the relationship between health and social determinants of health and how social determinants vary across world regions compared to 31% of physiotherapy students (*P* = 0.03). Additionally, almost 80% of the occupational therapy students considered that “relationship between access to clean water, sanitation, and nutrition on individual and population health” is a very important topic to learn in global health compared to 21% of physiotherapy students (*P* = 0.03) (Table 4).

Both physiotherapy and occupational therapy students (a total of 25 students) suggested additional topics that are important to learn in global health including (1) “understanding the different structures of health care around the world,” (2) “access to adequate healthcare services in less developed countries,” (3) “cultural perceptions of disability, work and health,” (4) “global health issues and social determinants of health,” (5) “language barrier and effective communication,” (6) “Potential cultural clash experiences of young immigrants, stereotyping based on religion, knowing the effects politics has on health in certain countries in the world,” (7) “the impact of climate change on the health of low socioeconomic classes,” (8) “specific education surrounding aboriginal people and the effects that their lifestyles have on them physically, psychologically, and emotionally,” and (9) “WHO millennium development goals.”

## 4. Discussion

Our study identified several knowledge and skill opportunities relevant to global health and health equity for rehabilitation sciences students. These needs overlap with other primary health professionals but differ between professions, suggesting a need for both interprofessional and intraprofessional prioritization for education and policy relevance. Overall, both occupational therapy and physiotherapy students demonstrated limited competencies in global health. Few items received scores over 60%.

Most participants in our survey were females that spoke two languages and came from families with high socioeconomic status. The students' sociodemographic profile is an important component for effective care in a global health context. The recent report by the Commission on Education of Health Professionals for the 21st century refers that in many countries the competencies of graduate students might not be aligned with the new challenges and the social, linguistic, and ethnic diversity of the populations [21]. Our findings confirm the results of the recent commission report which states that health professional students admitted in health programs come from higher social classes and dominant ethnic groups [21].

Regarding gender, our findings were consistent with those of Cockrell and Peplau [22, 23] that there is a predominance of females in health programs. Moreover, the Canadian Institute for Health Information (CIHI) 2009 report also found that occupational therapists had higher proportion of women in their workforce (92.0%) compared to various other health professions, such as physiotherapists (78.0%), pharmacists (59.2%), and physicians (34.7%) [24]. This evidence validates that females dominate the workforce of physiotherapy and occupational therapy in all provinces of Canada [24].

Gender balance in health systems is highly recommended, as a gender imbalance is a major obstacle for access to health care [25, 26]. There is extensive literature on “feminization” of parts of the health workforce and high concentration of women in health professions. This trend is directly related to globalization and it has consequences to global health. It can bring many consequences for health systems, such as practice location and practice hours. Female professionals are more likely to practice in urban areas rather than rural areas [27]. Globally, nearly one half of the population lives in rural areas and, according to the WHO, the health professional workforce shortage in rural areas is a worldwide problem that affects almost all countries [28]. The WHO also recommends admission policies to enroll students with a rural background in order to increase the probability of these students developing their practice in rural areas [28]. Moreover, a number of studies have documented that women work fewer hours in their professional careers than do men [29].

In our study, almost half of the physiotherapy student participants self-reported that they are able to speak at least two languages while half of the occupational therapy student participants reported that they are only able to speak one language. In addition, more than 60% of the physiotherapy participants reported self-perceived confidence related to language barriers and adverse impact on health and health care. According to the literature, one of the barriers to effective health care services is language [30, 31]. Multilinguistic knowledge and skills help health professionals to provide effective care for their clients. This finding is compatible with a similar survey about self-perceived knowledge of underserved population topics with family physician residents in United States (Wieland Survey) where they reported 61.0% had confidence in this topic [16]. From the service provider's perspective, language may be a barrier to the health professionals in understanding the information and beliefs regarding patients' health and address the difference or incongruence in the values of patients and physiotherapists and occupational therapists [32].

On the other hand, our result in relation to the confidence in the “relationship between income and health” differs from the Wieland study. More than 80% of the occupational therapy students assessed themselves as confident in the “relationship between income and health,” while 57.4% of US family physician residents surveyed reported confidence in the topic. We believe that this difference is related to the main role of the occupational therapists. The occupational therapy profession in itself has a crucial role in training and offering advice on occupational performance, including self-care and productive and recreational activities [26]. Their interventions are also very effective for reducing personal and societal costs of work-related injuries as well as reducing the duration of work disabilities [26]. Hence, knowledge in “relationship between income and health” is a crucial competence for occupational therapists in all settings and therefore their confidence in this area is not surprising.

Physiotherapy and occupational therapy students reported less confidence in “access to health care for low income nations,” “mechanisms for why racial and ethnic disparities exist,” and “racial stereotyping and medical decision making.” For these topics, our results were similar to the US students survey, where all of them scored less than 50% [16]. Socioeconomic status and racial disparities are important determinants of health outcomes [33, 34] and are extremely important for working with a global health perspective.

Overall, students from both programs reported less skill in “global health activity.” Physiotherapists and occupational therapists need to have extended knowledge and skills in global health in addition to their core professional training, to tackle the global burden of disease and disabilities in this multicultural world of the 21st century. The complexity of global health work demands physiotherapists and occupational therapists to have not only clinical and rehabilitation abilities, but also skill and knowledge regarding epidemiology, sociology, population health, geography, laws, and other disciplines. These disciplines are crucial for working in partnership with governmental and nongovernmental organizations.

Another point of emphasis is that in this globalized world occupational therapists and physiotherapists must build confidence to meet the demand and quality care of international citizens from all over the world, including immigrants and refugees, disabled people, victims of wars, and those who suffer from infectious diseases in order to provide equitable services. Furthermore, all the global health challenges will come to “knock on the door” of all health professionals wherever they are working in different settings sectors, such as clinics, rehabilitation centers, community health, or in hospitals.

### 4.1. Strengths and Limitations of the Study

This study begins to address important knowledge gaps that have appeared in global health literature and provides some global health relevant elements for the disciplines of physiotherapy and occupational therapy. It also highlights how future students of these disciplines can address the growing needs of global health effectively and equitably. Although the findings of this study are important, the results of this study should be interpreted with caution and should not be generalized for all populations because of the small sample size and the limited response rate. We used several strategies to improve the response rate such as e-mail and/or phone call communications with the coordinator and/or responsible for the health programs in the five universities in Ontario, in order to engage them in the research and two reminders were sent to the participants within two-week interval. Unfortunately, these strategies were not enough to minimize the low response rate already expected. Another limitation of the research was the availability of the survey only in English. Some health programs are offered only in French and even though we sent an e-mail invitation to the participants in French, it was not sufficient to motivate French speaking students to participate.

### 4.2. Implications for Future Research

Areas for further research include the following.

1. Assessing global health competencies with francophone students in Canada using a French version of the survey.
2. The use of qualitative and quantitative methods to evaluate students' learning needs in relation to global health knowledge, skills, and attitudes.
3. Assessing the range of issues that relates to global health that affects PT/OT practice during their local/international training settings.

### 4.3. Considerations for Action

Based on our findings and the current literature, considerations for action to improve global health education for rehabilitation students include the following.

- Implementing and building on existing inter-professional global health curriculum and mentorship programs into, for example, the open access interdisciplinary Refugees and Global Health e-Learning Program [35] and the Canadian Society of International Health Student Mentorship Program http://www.csih.org/en/about/job-opportunities/mentornet/.
- Considering targeted admission policies to recruit students from different ethnic and language backgrounds over a range of socioeconomic status, as there is evidence that workforce diversity may lead to improved health care equity [36].
- Providing opportunities for overseas global health training, seminars, and workshops that could improve students' knowledge and language skills, relevant to global health.

## 5. Conclusion

In conclusion, this paper expands our understanding of the emerging knowledge and skills that occupational therapy and physiotherapy students may need to provide fair and equitable health care more effectively. It provides an invitation for physiotherapy and occupational therapy educators and students to be more involved in emerging interdisciplinary global health initiatives and considerations for the rehabilitation profession. In this globalized 21st century, it is essential for all health professionals to tackle determinants of health (e.g., socioeconomic, environmental, and political factors) and develop competencies to work with other disciplines and to be globally interconnected to help reduce health inequalities. Therefore, improving global health knowledge and skills of occupational therapists and physiotherapists on global health competency is essential not only to prove care for local socially disadvantaged and disabled populations, but also to play leadership roles in the field of interdisciplinary global health.

## Figures and Tables

**Table 1:** Demographic characteristics of respondents (*N* = 166).

Variables	Number (percentage)	
	Physiotherapy students *n* = 68	Occupational therapy students *n* = 98
Sex		
Male	13 (19.1)	5 (5.1)
Female	55 (80.9)	93 (94.9)
Country (of birth)		
Canada	59 (86.8)	87 (88.8)
United States	0 (0.0)	1 (1.0)
Philippines	1 (1.5)	1 (1.0)
India	1 (1.5)	0 (0.0)
Honk Hong	3 (4.4)	3 (3.1)
Pakistan	0 (0.0)	1 (1.0)
Other	4 (5.9)	5 (5.1)
Mean age	26.47 (41)	24.91 (59)
Family background		
White	52 (76.4)	78 (79.6)
Chinese	7 (10.3)	7 (7.1)
South Asian	3 (4.4)	6 (6.1)
Black	0 (0.0)	1 (1.0)
Other	6 (8.8)	6 (6.1)
Parent's family income		
$20,001 to $30,000	8 (11.8)	1 (1.0)
$30,001 to $40,000	1 (1.5)	3 (3.1)
$40,001 to $50,000	4 (5.9)	3 (3.1)
$50,001 to $60,000	2 (2.9)	8 (8.2)
$60,001 to $70,000	4 (5.9)	4 (4.1)
$70,001 to $80,000	5 (7.4)	10 (10.2)
$80,001 or more	23 (33.8)	34 (34.7)
Do not know	21 (30.9)	35 (35.7)
Languages (spoken)		
One language	26 (38.2)	48 (49.0)
Two languages	31 (45.6)	38 (38.8)
Three languages	8 (11.8)	6 (6.1)
Four languages or more	3 (4.4)	6 (6.1)

**Table 2:** Physiotherapy and occupational therapy students' self-perceived knowledge in global health.

Domains of self-perceived knowledge	Physiotherapy students' score* (%)	Occupational therapy students' score* (%)
Language barrier and adverse impact on health and health care	65.67	57.65
Access to health care for low income nations	25.74	29.59
Relationship between income and health	71.32	76.53
Relationship between work and health	68.38	81.63
SEP and impact on health	64.71	67.86
Environmental health and socioeconomic position	47.06	53.06
Relationship between housing and health status	55.15	54.12
SEP and food security confidence	54.41	48.47
Health outcome discrepancies among different groups in Canada	39.71	40.82
Mechanisms for why racial and ethnic disparities exist	32.35	31.12
Racial stereotyping and medical decision making	36.76	38.54
Gender and access to health care	42.65	40.82

*Average perceived knowledge and skills percentage for each domain varied for item scale between 0 and 1 (item scale [0-1]: 0 = not at all confident; 0.5 = somewhat confident; 1 = very confident).

**Table 3:** Perceived global health skills for physiotherapy and occupational therapy students guided by the CanMEDS framework.

Skills	Physiotherapy students' score* (%)	Occupational therapy students' score* (%)
Communication skills	60.00	54.34
Listening skills	76.17	72.68
Able to understand patient with different background skills	54.04	56.63
Address team disagreement skills	52.69	42.89
Discuss sensitive issues skills	49.62	48.95
Identify needs skills	63.28	56.84
Helping patients achieve realistic goals skills	55.68	47.83
Working in a team skills	55.47	53.95
Clinical competency Skills	69.62	67.11
Keep up to date in global health skills	50.47	53.16
Active in global health skills	33.09	33.42

*Average perceived knowledge and skills percentage for each domain varied for item scale between 0 and 1 (item scale [0-1]: for negative questions: 1 = strongly disagree, 0.75 = disagree, 0.50 = neutral, 0.25 = agree, 0 = strongly agree); for positive questions (1 = strongly agree, 0.75 = agree, 0.50 = neutral, 0.25 = disagree, 0 = strongly disagree).

**Table 4:** Learning needs in global health for physiotherapy and occupational therapy students in Ontario, Canada.

Learning needs in global health	Physiotherapy *N* (%)	Occupational therapy *N* (%)	Pearson chi-square
Health risks associated with travel and migration, with emphasis on possible risks and appropriate management, including referrals			
Not important	0 (0)	0 (0)	
Somewhat important	16 (64)	9 (36)	0.12
Neutral	7 (28)	18 (72)	
Important	24 (40.7)	35 (59.3)	
Very important	16 (40)	24 (60)	
Extremely important	4 (36.4)	7 (63.6)	
Knowledge about how travel and trade contribute to the spread of communicable diseases			
Not important	0 (0)	0 (0)	
Somewhat important	9 (42.9)	12 (57.1)	0.86
Neutral	9 (33.3)	18 (66.7)	
Important	27 (45.8)	32 (54.2)	
Very important	15 (45.8)	20 (57.1)	
Extremely important	7 (38.9)	11 (61.1)	
Relationship between health and social determinants of health, and how social determinants vary across world regions			
Not important	0 (0)	0 (0)	
Somewhat important	3 (75)	1 (25)	0.03
Neutral	5 (62.5)	3 (37.5)	
Important	28 (53.8)	24 (46.2)	
Very important	17 (32.7)	35 (67.3)	
Extremely important	15 (31.3)	33 (68.8)	
Relationship between access to clean water, sanitation, and nutrition on individual and population health			
Not important	2 (50)	2 (50)	0.03
Somewhat important	4 (40)	6 (60)	
Neutral	5 (55.6)	4 (44.4)	
Important	18 (36)	32 (64)	
Very important	9 (20.9)	34 (79.1)	
Extremely important	30 (62.5)	18 (37.5)	
Understand the relationship between health and human rights			
Not important	1 (50)	1 (50)	0.28
Somewhat important	2 (66.7)	1 (33.3)	
Neutral	4 (50)	4 (50)	
Important	18 (43.9)	23 (56.1)	
Very important	14 (27.5)	37 (72.5)	
Extremely important	29 (47.5)	32 (52.5)	
Knowledge about how global health institutions (e.g., WHO, other United Nations agencies, and global institutions) influence health in different world regions through funding and policy			
Not important	1 (33.3)	2 (66.7)	0.74
Somewhat important	2 (50)	2 (50)	
Neutral	6 (54.5)	5 (45.5)	
Important	24 (46.2)	28 (53.8)	
Very important	22 (35.5)	40 (64.5)	
Extremely important	12 (36.4)	21 (63.6)	
